# Dietary inflammatory index and inflammatory markers in Brazilian adolescents

**DOI:** 10.11606/s1518-8787.2024058005485

**Published:** 2024-07-10

**Authors:** Renata de Sousa Gomes, Poliana Cristina de Almeida Fonseca Viola, Roberta Rejane Santos de Carvalho, Nitin Shivappa, James R. Hebert, Ana Karina Teixeira da Cunha França, Carolina Abreu de Carvalho

**Affiliations:** I Universidade Federal do Maranhão Programa de Pós-graduação em Saúde Coletiva São Luís MA Brasil Universidade Federal do Maranhão. Programa de Pós-graduação em Saúde Coletiva. São Luís, MA, Brasil; II Universidade Federal do Piauí Departamento de Nutrição Teresina PI Brasil Universidade Federal do Piauí. Departamento de Nutrição. Teresina, PI, Brasil; III University of South Carolina Arnold School of Public Health Department of Epidemiology and Biostatistics Columbia SC United States of America University of South Carolina. Arnold School of Public Health. Department of Epidemiology and Biostatistics. Columbia, SC, United States of America; IV Universidade Federal do Maranhão Departamento de Ciências Fisiológicas Programa de Pós-graduação em Saúde Coletiva São Luís MA Brasil Universidade Federal do Maranhão. Departamento de Ciências Fisiológicas. Programa de Pós-graduação em Saúde Coletiva. São Luís, MA, Brasil; V Universidade Federal do Maranhão Departamento de Saúde Pública Programa de Pós-graduação em Saúde Coletiva São Luís MA Brasil Universidade Federal do Maranhão. Departamento de Saúde Pública. Programa de Pós-graduação em Saúde Coletiva. São Luís, MA, Brasil

**Keywords:** Adolescents, Diet, Chronic Inflammation, Markers Inflammatory, Food Consumption

## Abstract

**OBJECTIVE:**

To evaluate the association between the inflammatory potential of the diet measured by the energy-adjusted diet inflammatory index (E-DII) and inflammatory markers in adolescents.

**METHODS:**

This cross-sectional study was conducted among 518 adolescents aged 18 and 19 years from São Luís, Maranhão, Brazil in 2016. A semiquantitative food frequency questionnaire (FFQ) was used to assess dietary intake from which E-DII scores were calculated to determine the inflammatory potential of the diet. The associations between E-DII and inflammatory markers (hs-CRP, IL-6, IL-4, TNF-α, and IFNγ) were analyzed using multivariable linear regression. The variables included in the adjusted model were identified using the directed acyclic graph.

**RESULTS:**

The diet of these adolescents was mostly pro-inflammatory; mean E-DII score was 1.71 and ranged from -2.44 to 5.58. Higher E-DII scores were positively associated with higher levels of IFNγ in the adjusted analysis (Adjusted Coef.: 1.19; 95%CI: 0.36–12.04). We observed no associations between E-DII and other inflammatory markers (hs-CRP, IL-6, IL-4, TNF-α). Study results indicate that E-DII is useful in evaluating the inflammatory potential of the diet of Brazilian adolescents.

**CONCLUSIONS:**

Cross-sectionally E-DII scores were positively associated with IFNγ concentrations. Future research should examine the association between changes in E-DII scores and levels of inflammatory markers longitudinally.

## INTRODUCTION

Chronic low-grade systemic inflammation, defined by the persistence of inflammatory processes beyond their physiological function, is characterized by the continuous elevation of inflammatory markers. This chronic pro-inflammatory state induces oxidative stress, insulin resistance, and vascular dysfunction with a consequent increase in the risk of metabolic diseases, atherosclerosis, and cancer. Indeed, this state of chronic inflammation is considered the main mechanism involved in the development of most noncommunicable diseases (NCDs)^1^and autoimmune diseases^2^.

Studies indicate the role of dietary patterns and consumption of specific foods or nutrients on the modulation of the inflammatory process. A diet rich in ultra-processed foods, with excess saturated fat and sugar and low in fiber, has been associated with chronic, low-grade inflammation^3,4^. On the other hand, dietary patterns that emphasize the frequent consumption of fruits and vegetables, whole foods, fish, and vegetable oil, restricting the intake of saturated fat, red meat, and ultra-processed foods, are related to lower levels of inflammatory markers^5,6^.

The diet inflammatory index (DII®) was created to assess the potential inflammatory diet of populations, which is a score obtained from the sum of the anti-inflammatory and pro-inflammatory effects of 45 dietary parameters, such as macronutrients, fiber, cholesterol, fatty acids, flavonoids, some vitamins, and minerals, as well as some spices^7^. Subsequently, the energy-adjusted diet inflammatory index (E-DII^TM^) was developed^8^.

Over 40 articles have been published on the association between the DII, E-DII, and children’s diet inflammatory index (C-DII) with inflammatory markers in children and across a wide age range in adults, suggesting its validity in detecting the dietary inflammatory potential^9^. However, few studies have proposed to evaluate this association in younger age groups, such as adolescents^14^, and none of them in Brazilians.

Verifying the association of the DII /E-DII with inflammatory markers is essential since it indicates whether the DII can capture the inflammatory potential of the diet in younger populations and countries other than those where this association has been verified so far. In this context, this study evaluated the association between E-DII and inflammatory markers in adolescents aged 18 and 19 years from São Luís, Maranhão, Brazil.

## METHODS

### Location, Study Design, and Sampling

This cross-sectional study was conducted within a cohort developed in Brazil in the municipality of São Luís, state of Maranhão (MA). The initial cohort study in São Luís included live births from 10 hospitals, both public and private, from March 1997 to February 1998. During the second follow-up, children aged seven to nine years were evaluated from 2004 to 2006. Then, the third follow-up occurred when the participants were aged 18 and 19 years. Our study used data only from the third phase of the cohort, conducted in 2016.

Adolescents of the cohort were searched via lists of public and private schools, Military Enlistment Boards, registers of the Municipal Department of Children and Social Assistance, telephone numbers recorded in the previous phase of the cohort, and social media. A total of 684 participants from the original cohort attended the interview. Of these, 533 provided blood samples in which their inflammatory markers were collected.

**Figure 1:**
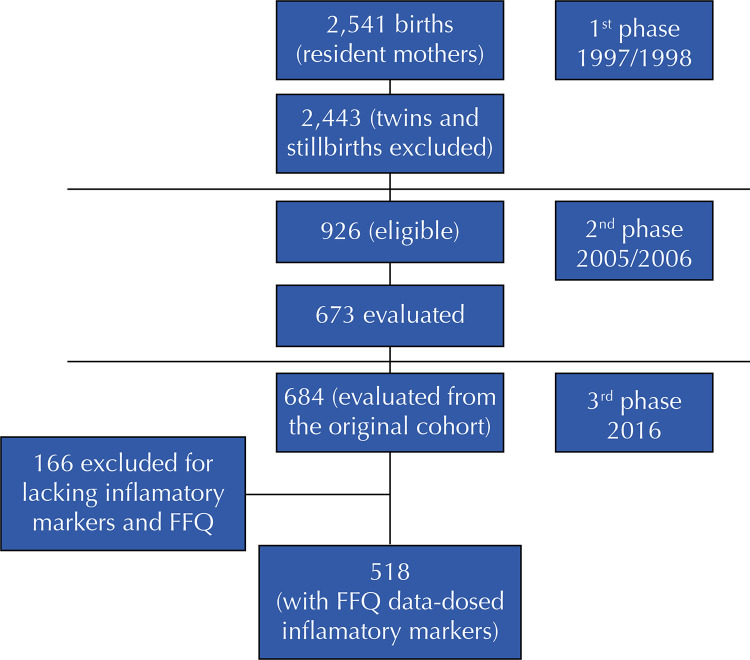
Flowchart of the birth cohort of São Luís 1997/98. São Luís, Maranhão, Brazil, 2016.

The study’s final sample accounted for 518 participants from the original cohort, including only adolescents who responded to the dietary survey and had their inflammatory markers evaluated (Figure 1). Pregnant adolescents or adolescents with physical limitations that could compromise the anthropometric evaluation were excluded from the study.

Subtitle: FFQ – Food frequency questionnaire.

### Data Collection Procedures

Data collection was conducted at the Federal University of Maranhão (UFMA) and was performed by trained health professionals. The collected data were recorded using the software Research Electronic Data Capture (REDcap. https://www.projec t-redcap.org/). All the interviewers and evaluators who participated in the research were highly trained. Data were submitted to quality control procedures to look for errors and inconsistencies in variables.

### Inflammatory Markers Concentrations

A volume of 40 mL of blood from the cubital vein was obtained, centrifuged, and stored in a freezer at −80°C. The pro-inflammatory markers were analyzed using the Multiplex MAP Human Cytokine Kit technology, manufactured by Merck (Darmestádio, Germany), and were evaluated as continuous numerical variables^17^. The inflammatory markers evaluated in this study were: ultrasensitive C-reactive protein (us-CRP) in ng/mL, tumor necrosis factor-alpha (TNF-α) in pg/mL, Interleukin-4 in (IL-4) pg/mL, interleukin-6 (IL-6) in pg/mL, and interferon-gamma (IFNγ) in pg/mL.

### Food Intake

To evaluate food intake, an adapted version of a Food Frequency Questionnaire (FFQ) containing 118 food items was administered by a nutritionist, referring to the consumption of the last 12 months, developed by Schneider et al.^18^ (2016). This questionnaire was adapted for use in this research and validated by Bogea et al.^19^ (2021). Computer devices were used to apply the FFQ, which showed the food sections on the screen. Each food item contained eight response options for estimating the consumption of each specific food, namely: Never or < 1×/month; 1 – 3×/week; 2 – 4×/week; 5 – 6×/week; 1×/day; 2 – 4×/day; and 5×/day. Regarding portion sizes, adolescents were asked if they consumed an average amount, a larger amount (1.5 times the average percentage), or a smaller amount (half the part).

To quantify food intake and determine DII parameters, the Stata® statistical software version 14 was used. First, the frequency of consumption of each food item and the size of the reported portion were transformed into annual consumption. Then the daily consumption of each item was determined. The analysis of consumed nutrients was performed based on the mean values established by the nutritional composition table of the *Pesquisa de Orçamentos Familiares* (POF – Household Budget Survey)^20^ and the Brazilian food composition table TACO^21^. Nutrients not included in the TACO were determined only by the POF table^20^. For the analysis of flavonoids and carotenoids, the complementary tables of the TBCA food composition table were used as references^22,23^. For the nutrients that are not available in the Brazilian food composition tables, the North American table (United States Department of Agriculture – USDA) was used; i.e., for alcohol and caffeine.

### Dietary Inflammatory Index

The DII assesses the inflammatory potential of the diet and was developed by Shivappa et al.^7^(2014) based on an extensive literature review that evaluated the effect of 45 specific dietary parameters, including energy, macronutrients and some micronutrients, and spices on specific inflammatory markers, including CRP, IL-1β, IL-4 IL-6, IL-10, and TNF-α. Each dietary parameter received an inflammatory score based on this literature review. For example, macronutrients presented pro-inflammatory scores, whereas parameters such as omega 6 and omega 3 fatty acids, fiber, flavonoids, ginger, and spices showed anti-inflammatory scores.

To standardize the measurement units and avoid arbitrary values, the authors gathered a representative food consumption database from 11 countries in different regions of the world^7^. From this database, means and reference standard deviations were generated for each food parameter. The food consumption data of our study were standardized by the mean and standard deviation of reference of each food parameter that composes the DII. These standardized values were multiplied by the inflammatory effect scores found in the DII literature review, resulting in the DII of each dietary parameter. Finally, to obtain the total DII of each person, scores of all dietary parameters consumed were combined. The study by Shivappa et al.^7^(2014) provides further information on the methodology and explanation of the DII.

To determine the DII in this study, 40 dietary parameters included in the FFQ were used: energy, carbohydrate, protein, lipid, fiber, iron, retinol, thiamine, riboflavin, niacin, vitamin C, cholesterol, saturated fatty acids, monounsaturated fatty acids, polyunsaturated fatty acids, including omega 6 and omega 3 fatty acids, selenium, folic acid, vitamin D, vitamin B_12_, vitamin B_6_, vitamin E, magnesium, zinc, β-carotene, flavanols, flavonols, flavones, anthocyanins, flavonones, caffeine, alcohol, garlic, onion, rosemary, ginger, saffron, oregano, and trans-fat. In total, five dietary parameters were not evaluated in the FFQ and, therefore, were not included in the DII estimate: pepper, turmeric, eugenol, green/black tea, and isoflavone.

The E-DII was used to control total energy intake, which refers to the DII estimated per 1,000 kcal^8^. The E-DII is calculated similarly to the DII, except that all dietary exposures are expressed per 1,000 kcal/day and the reference database is also expressed per 1,000 kcal/day.

### Sociodemographic, Economic, and Lifestyle data

Participants answered a semi-structured questionnaire containing sociodemographic (sex, age, skin-color/ethnicity), economic (monthly household income *per capita*), and lifestyle (smoking habit and alcohol consumption) data. Smoking habit was categorized as yes or no, and alcohol consumption was categorized as: never or once a month or less; two to four times a month; and two or more times a week. Skin-color/ethnicity was self-reported, according to the criteria established by the Brazilian census, denoting perceived phenotype (physical appearance) and not origin (ancestry). Monthly household income *per capita* was collected in Brazilian *reais* and categorized into tertiles.

### Physical Activity

For the classification of the level of physical activity, a 24-hour physical activity recall was used, adapted from the Self-Administered Physical Activity Checklist (SAPAC)^24^. This index is a result of the multiplication of the time spent in each physical activity by the number of days performed, and its objective is to obtain the quantification of weekly physical activity. The expenditure on physical activity is defined in metabolic equivalents (METs). To estimate metabolic expenditure, the METs of each type of activity were obtained by the Compendium of Physical Activities^25^; moderate activities were considered as 4.5 to 6 METs expenditure and intense activity as higher than 6 METs. The recommendation proposed by the World Health Organization (WHO) of at least 75 minutes of intense physical activity or 150 min of moderate activity per week was used^26^.

### Anthropometric Evaluation

For the classification of nutritional status, the Body Mass Index (BMI = weight (kg)/height (m)^2^) was used for age and body fat percentage (BF%). The BMI for age estimated in z-score was classified following the growth curves recommended by the WHO^27^. Weight was evaluated on a high-precision scale coupled to the air displacement plethysmography equipment BOD POD Gold Standard of the brand COSMED® (*COSMED Metabolic Company,* Rome, Italy). Height was measured using the AlturaExata® portable stadiometer (Belo Horizonte, Brazil). The BF% was measured using the air displacement plethysmography method, using the BOD POD®. The Williams criterion was used for the %BF classification. Thus, values lower than 25% for males and 30% for females were considered as normal BF%; and values greater than 25% for males and 30% for females were considered as high BF%^28^. The BMI and BF% classifications were used only for the characterization of the sample. Subsequently, BMI and BF% were used in the association analyses as continuous variables.

### Blood Pressure

Arterial blood pressure (BP) was obtained using the mean of three measurements after at least five minutes at rest, with an interval of one minute between each measurement, in the sitting position, with the dominant arm resting on a support, positioning the artery at the same level as the heart. The Omron HEM 742INT® blood pressure monitoring device was used (São Paulo, Brazil). BP was classified based on the American Guideline of Clinical Practice for Screening and Treatment of Hypertension in Children and Adolescents, which establishes BP values for adolescents over 13 years of age, classified as 1) Normal BP: < 120/80 mm Hg; 2) Elevated BP: 120/80 to 129/80 mm Hg; and 3) Hypertension: ≥ 130/80 mm Hg^29^.

### Statistical Data Processing and Analysis

The data were analyzed in the Stata® statistical software version 14. The Kolmogorov-Smirnov test and the analysis of histograms and box-plot graphs were used to verify the normality of numerical variables. In this analysis, the presence of outliers in the variable IFNγ were observed. Therefore, values above 50 pg/mL (higher than the 99th percentile) were excluded in the tests that included this variable to improve the analyses performance. In total, 34 individuals were excluded, representing 6.5% of the sample.

Categorical variables were presented by frequency and percentage. Numerical variables were presented using the mean and standard deviation or median and interquartile intervals. Pearson’s correlation coefficients were estimated to measure the correlation between E-DII score and inflammatory markers.

The E-DII was analyzed as a categorical variable in tertiles, the lowest being the most anti-inflammatory. Pearson’s Chi-square test was used to compare socioeconomic and lifestyle characteristics within the E-DII tertiles for categorical variables. The comparison of means was performed by analysis of variance (ANOVA) for continuous variables.

**Figure 2:**
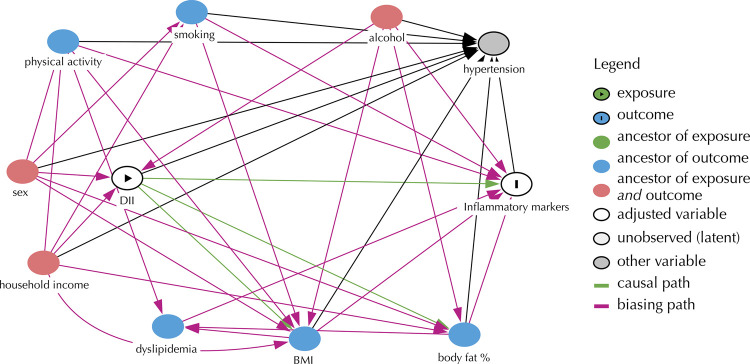
Directed acyclic graph (DAG) of the relationship between E-DII and inflammatory markers.

Multivariable linear regression analysis was used to evaluate the association between E-DII (exposure) score and inflammatory markers (outcome). To select the adjustment variables of the linear regression analysis, a directed acyclic graph (DAG) was used. The DAG codifies causative relationships between variables and allows the identification of a minimum set of adjustment variables necessary to study the association between E-DII and inflammatory markers (Figure 2). Therefore, using DAG helps to consider the confounding variables according to the literature evidence, minimizing confounding bias and avoiding overadjustment of the variables^30,31^.

The variables indicated for the adjustment of the multivariable analysis included sex, household income, and alcohol consumption. It is important to consider that obesity measured both by BMI and percentage of fat, despite being variables known to influence inflammatory markers, are not considered confounding factors in the association studied in this work since they do not cause exposure (E-DII). For this reason, these variables were not included in the adjustment for multivariable analysis. For all analyses, the significance level was set at 5%.

### Ethical Aspects

This study was conducted following the guidelines proposed by Declaration of Helsinki and all procedures involving human subjects/patients were approved by the Research Ethics Committee of the University Hospital of the Federal University of Maranhão (HU-UFMA), No. 1.302.489. All participants signed an informed consent form.

## RESULTS

Of the adolescents evaluated, 62.2% were male. The E-DII scores ranged from −2.4 to 5.6 points, with a mean of 1.71 ± 1.4 points. The median household income *per capita* was 374.7 Brazilian *reais* (IQR: 197 – 621.6). Most adolescents were not smokers (96.1%), 48.8% did not meet the physical activity recommendation, 64.9% reported being Mixed-race, and 18.3% and 25.5% suffered from prehypertension and hypertension, respectively. Moreover, mean BMI was 22.3 ± 4.3 Kg/m^2^ and BF% was 18.3 ± 11.5%. Regarding the consumption of alcoholic beverages, 77.5% reported not consuming or consuming at most once a month and 17.9% reported consuming two to four times a month (Table 1). We observed that 17.8% of adolescents were overweight and 19.9% had high BF% (data not shown).

**Table 1:** Socioeconomic, anthropometric, and lifestyle characteristics according to the tertile of the E-DII, São Luís – MA, Brazil, 2016.

Characteristic	All (%)	E-DII tertiles			p-value

		T1	T2	T3	
Sex					0.110
Female	37.84	30.61	30.61	38.78	
Male	62.16	35.09	35.09	29.81	
Color					0.895
White	16.28	33.33	29.76	36.90	
Black	18.60	35.42	33.33	31.25	
Mixed-race/Yellow	65.12	32.74	34.52	32.71	
Household income *Per capita* (median in *reais*)	374.7	100.0	372.4	845.0	0.422
Interquartile range	197–621.7	0–220.0	266.7–442.5	555.0–1320	
Physical activity (%)					0.156
Met recommendations	51.17	29.08	34.66	36.25	
Below recommendations	48.83	36.88	32.32	30.80	
Smoking habit					0.198
No	96.14	32.93	34.14	32.93	
Yes	3.86	45.00	15.00	40.00	
Alcohol consumption					0.506
Never or once a month or less	77.54	34.26	34.26	31.49	
Two to four times a month	17.97	29.35	29.35	41.30	
Two or more times a week	4.49	30.43	34.78	34.78	
BMI (mean)	21.78	22.28	21.61	21.44	0.099
Standard deviation	3.81	4.26	3.56	3.55	
% Body fat (mean)	18.21	18.28	16.91	19.47	0.089
Standard deviation	10.73	11.53	9.96	10.54	
Arterial blood pressure					0.479
Normal	56.18%	30.93	34.36	34.71	
Pre-hypertension	18.34%	37.68	28.99	33.33	
Arterial hypertension	25.48%	34.83	37.08	28.09	

E-DII: energy-adjusted dietary inflammatory index; BMI: body mass index.

Table 1 shows the socioeconomic, anthropometric, and lifestyle characteristics according to the E-DII tertiles. We noted no significant differences between the E-DII tertiles for any socioeconomic, anthropometric, and lifestyle variables analyzed.

Table 2 shows the mean consumption of dietary parameters according to the E-DII tertiles. The intake of total fat, saturated fat, trans fat, polyunsaturated fatty acids, monounsaturated fatty acids, omega 6 fatty acids, and vitamin A was significantly higher across E-DII tertiles. In contrast, consumption of fiber, pyridoxine, vitamin C, magnesium, flavonols, flavonones, β-carotene, garlic, and onion were significantly lower with the increase of E-DII tertiles.

**Table 2:** Distribution of dietary parameters according to E-DII tertiles, São Luís – MA, Brazil, 2016.

Nutrients	E-DII tertiles			p-value

	T1	T2	T3	
Energy (kcal)	2,999.86	3,133.78	3,373.2	0.09
Carbohydrates (g)	469.87	478.81	501.43	0.45
Proteins (g)	114.01	112.07	109.56	0.79
Total fats (g)	73.80	85.58	103.24	< 0.01
Saturated fats (g)	27.23	31.59	39.04	< 0.01
Trans fats (g)	4.33	5.16	5.40	0.01
Cholesterol (mg)	440.63	437.93	462.20	0.73
Monounsaturated fatty acids (g)	22.96	26.07	31.26	< 0.01
Polyunsaturated fatty acids (g)	13.31	14.56	17.21	< 0.01
Omega-3 (g)	1.01	0.97	0.99	0.86
Omega-6 (g)	8.69	9.50	11.12	0.01
Fibers (g)	45.67	41.03	33.11	< 0.01
β-carotene (μg)	1,717.84	1,273.15	1,038.65	< 0.01
Thiamine (mg)	1.59	1.66	1.67	0.59
Riboflavin (mg)	2.02	2.08	2.31	0.02
Niacin (mg)	19.22	18.61	19.99	0.59
Pyridoxine (mg)	2.29	1.97	1.98	<0.05
Folic acid (μg)	562.09	564.96	553.93	0.91
Vitamin B_12_ (µg)	14.84	14.66	15.36	0.82
Vitamin A (µg)	1,648.39	1,569.04	1,667.05	< 0.01
Vitamin C (mg)	211.17	159.40	138.22	< 0.01
Vitamin D (μg)	0.93	0.89	0.89	0.90
Vitamin E (mg)	7.89	7.26	6.82	0.05
Iron (mg)	15.80	15.34	14.03	0.14
Zinc (mg)	15.78	15.25	14.43	0.28
Selenium (μg)	108.90	108.75	114.75	0.54
Magnesium (mg)	408.21	365.88	331.97	0.01
Flavonols (mg)	46.84	36.01	16.90	< 0.01
Flavones (mg)	1.73	1.33	1.15	0.45
Flavonones (mg)	11.49	5.93	6.28	< 0.01
Flavanols (mg)	13.54	11.63	10.24	0.50
Anthocyanidins (mg)	28.81	22.37	20.17	0.26
Caffeine (g)	0.11	0.09	0.11	0.50
Alcohol (g)	2.23	1.46	2.01	0.29
Garlic (g)	5.22	3.74	2.62	< 0.01
Rosemary (mg)	0.02	0.01	0.01	0.40
Ginger (g)	0.02	0.02	0.03	0.99
Oregano (mg)	0.08	0.10	0.09	0.76
Saffron (g)	0.03	0.04	0.07	0.32
Onion (g)	23.43	15.60	8.31	< 0.01

E-DII: Energy-adjusted dietary inflammatory index; kcal: kilocalories; g: grams; mg: milligram; μg: micrograms.

Among the inflammatory markers, IFNγ was positively associated with E-DII tertiles. Adolescents within the third E-DII tertile (most pro-inflammatory) had higher IFNγ values than those of the other tertiles (p = 0.03). None of the other inflammatory markers were associated with E-DII tertiles (Table 3).

**Table 3:** Distribution of inflammatory markers according to E-DII tertiles, São Luís – MA, Brazil, 2016.

Inflammatory markers	E-DII tertiles			p-value

	T1	T2	T3	
CRP	0.25	0.26	0.21	0.69
IL-6	5.63	4.35	2.72	0.18
IL-4	28.69	29.12	60.70	0.33
TNF-α	6.65	5.92	6.22	0.24
IFNγ	8.86	8.94	11.35	0.03

E-DII: Energy-adjusted dietary inflammatory index; CRP: C-reactive protein; IL-6: Interleukin 6; IL-4: Interleukin 4; TNF-α: tumor necrosis factor-α; IFNγ: interferon-γ.

In the crude and adjusted multivariable linear regression analysis, E-DII showed no association with us-CRP, IL-6, IL-4, and TNF-α (Table 4). The IFNγ showed a significant association with the E-DII (Adjusted Coef.:1.19; 95%CI: 0.36–12.04), indicating that for each 1 unit increase in the E-DII, a 1.19 pg/mL increase in INFy was noted.

**Table 4:** Crude and adjusted analysis of the association between inflammatory markers and E-DII score, São Luís – MA, Brazil, 2016.

	Coef. Crude	95%CI	p-value	Coef. Adjust.	95%CI	p-value
CRP	-0.01	-0.05 – 0.03	0.56	-0.03	-0.09 – 0.02	0.27
IL-6	-0.80	-1.73 – 0.13	0.09	-1.09	-2.32 – 0.14	0.14
IL-4	8.77	-5.94 – 23.45	0.24	18.64	-7.23 – 44.51	0.16
TNF-α	-0.10	-0.36 – 0.15	0.43	-0.13	-0.51 – 0.25	0.50
IFNγ^a^	1.03	0.41 – 1.65	0.001	1.19	0.36 – 12.04	< 0.01

Coef.: coefficient; E-DII: energy-adjusted dietary inflammatory index; CI: confidence interval; CRP: C-reaction protein.

^a^ The analysis was performed excluding outliers, totaling n = 476 adolescents.

## DISCUSSION

To the best of our knowledge, this is the first study to evaluate the association between E-DII score and inflammatory markers in Brazilian adolescents. We observed a high E-DII mean in the studied sample, and this index was associated with inflammation in adolescents, specifically by the rise in the IFNγ pro-inflammatory marker. We observed that INFγ increases with E-DII score, indicating a pro-inflammatory diet.

The IFNγ is a primary pro-inflammatory cytokine in the acute and, mainly, chronic inflammatory process, which considerably induces the production of macrophages from other inflammatory mediators, such as TNF-α. In macrophages, the production of TNF-α usually promotes the release of many other inflammatory mediators, including IL-6^32^. Moreover, IFNγ is mainly produced by type 1 (Th1) lymphocytes, and cytokines produced by these cells suppress other anti-inflammatory cytokines produced by Th2 lymphocytes, such as IL-4, IL-5, IL-10, and IL-13^29^. The literature indicates that long-term increased IFNγ values are involved in developing autoimmune and neurodegenerative diseases^32^.

We observed no associations in the adjusted analysis between E-DII and the other inflammatory markers, including hs-CRP, IL-6, IL-4, and TNF-α, nor in the distribution of demographic and socioeconomic characteristics across E-DII tertiles. These results may be related to the time of exposure to foods that modulate inflammation, which may not have been sufficient since the group is young, aged 18 and 19 years.

We believe that the high inflammatory potential of the diet of these adolescents may not yet have affected the systemic outcome for chronic markers that may change later. However, the association of E-DII with IFNγ observed in this study indicates that the adolescent diets’ pro-inflammatory effects are already impacting their serum inflammatory state since IFNγ is a regulator of acute and chronic inflammatory processes and, among chronic inflammatory markers, is one of the first to change, inducing a cascade of changes in other inflammatory markers^32^.

Other studies have also observed no association between DII and CRP in children and adolescents, possibly due to lower CRP levels in the individuals evaluated at these younger ages^14^. On the other hand, in studies with adults with a higher proportion of individuals with increased serum CRP levels, this association could be observed^34^. The CRP increase is due to increases of other inflammatory cytokines, mainly IL-6 and, to a lesser extent, IL-1 and TNF-α^35^. In our study, no individual in the group presented with hs-CRP levels that indicated cardiovascular risk (> 3 mg/L), signaling a population with low levels of chronic inflammation. Therefore, in our study, the absence of adolescents with high CRP suggests that the pro-inflammatory diet have not yet resulted in more drastic chronic changes in inflammatory markers among this population.

The diets of these adolescents aged 18 and 19 were generally pro-inflammatory, highlighting a food pattern that is harmful to health, with E-DII values ranging from −2.44 to 5.58, with an average of 1.71. Other studies conducted with adolescents whose ages ranged from 10 to 19 years also reported pro-inflammatory DII means^14,16,36^; however, the mean obtained in our research was one of the highest ever reported. In a population study conducted in Brazil based on HBS data (2008-09), the mean DII among adolescents aged 10 to 19 years (1.04 ± 1.44), was higher compared to other age groups^36^, yet this mean is lower than that found in our study. The less healthy dietary pattern among adolescents, rich in ultra-processed foods with high fat, sodium, and sugar content and reduced in fiber^37^, helps explain the higher mean E-DII in this group compared with the adult population.

Consumption of total fat, saturated fat, and trans-fat was higher as E-DII increased. On the other hand, higher E-DII values were associated with lower intake of anti-inflammatory parameters, such as fiber, pyridoxine, vitamin C, magnesium, flavonols, flavonones, β-carotene, garlic, and onion. These results indicate the impact of the profile of consumed foods and nutrients on the E-DII. Therefore, the need to adhere to a diet rich in nutrients and foods with anti-inflammatory effects in adolescence is reinforced to obtain a lower E-DII and thus contribute to the prevention of chronic inflammation and NCDs^38^.

It is noteworthy that Maranhão holds the second lowest prevalence of ultra-processed food consumption among Brazilian states, in addition to presenting one of the lowest rates of overweight. Therefore, preventive measures should be implemented promptly to limit the growth of ultra-processed food consumption, which also shows a high pro-inflammatory potential.

We emphasize that our study presents limitations. First, the cross-sectional design limits causal inference since the temporality of the diet’s effect on outcomes cannot be established. This would require longitudinal studies. However, we highlight the use of a FFQ that sought to evaluate the frequency of food intake in the last 12 months, which is more likely to reflect a habitual diet in the period before the measurement of inflammatory markers. Second, using FFQ as a standard for assessing food intake may lead to some limitations, resulting from overestimation or underestimation of certain foods due to bias in memory and perception of food groups. Third, since it is a semi-quantitative FFQ, we must consider a limitation in the quantification of nutrients due to the imprecision of size portion estimations, which typically happens in this type of FFQ.

The five dietary parameters not included in the E-DII estimate of our study also present anti-inflammatory characteristics (pepper, turmeric, eugenol, green/black tea, and isoflavone). Therefore, their absence may have contributed to a more pro-inflammatory mean in the sample. However, most of the parameters not evaluated are seasonings (pepper and turmeric) or foods (green/black tea) and substances such as eugenol that adolescents may consume in low amounts. Despite the non-inclusion of these five parameters, the literature shows that other studies commonly cannot evaluate all 45 DII parameters. Our study assessed the highest number of parameters among those published with adolescents, while other studies used only 25^15^ to 31 parameters^14,16^. Finally, we highlight that, although it was necessary to exclude values of IFNγ considered implausible, when comparing the individuals included with the excluded, we found no significant differences according to sex, alcohol consumption, and household income (confounding variables in the current study).

We consider it relevant that dietary policies be targeted at adolescents more incisively since they tend to have poor food consumption. We also highlight the need of measuring the impacts and changes caused by the COVID-19 pandemic on the food consumption of populations, especially adolescents. These impacts should be also considered when implementing policies.

As strengths of this study, we can highlight the use of DAG to construct a theoretical model that allowed selecting the variables to adjust the model, considering potential confounding factors of the association studied. We used a validated semiquantitative FFQ capable of capturing the usual diet of adolescents. Our study also evaluated a high number of E-DII dietary parameters and a wide range of inflammatory markers. Finally, this is the first study to verify the association between DII and inflammatory markers in Brazilian adolescents, indicating that this index can be used in adolescents with similar characteristics to reflect the effect of diet on outcomes associated with inflammation.

## CONCLUSION

The results of our study pointed to a high mean E-DII score among adolescents from São Luís-MA and the association of a more pro-inflammatory diet with the elevation of the inflammatory marker IFNγ. This result demonstrates that E-DII can be used to identify individuals at risk of chronic inflammation even at an early age, as observed in the adolescents of this study. However, we do not exclude the need to make changes in the index to achieve a more sensitive performance between adolescents. It is worth considering a cultural adaptation since the spices that compound the index are not part of the food habits in many countries, including Brazil.

The E-DII was associated with a chronic inflammatory marker, IFNγ, suggesting the effect of diet on the health of the studied group. Therefore, establishing actions to promote an adequate and healthy diet among adolescents with a focus on the inflammatory potential of the diet is essential to prevent systemic inflammatory changes and, consequently, the onset of chronic non-communicable diseases.
